# *In vitro* and *in vivo* efficacy of the antimycobacterial molecule SQ109 against the human pathogenic fungus, *Cryptococcus neoformans*

**DOI:** 10.1371/journal.pntd.0013837

**Published:** 2025-12-16

**Authors:** Nour M. Alkashef, Ehab A. Salama, Ramu Anandakrishnan, Tony R. Hazbun, Micah V. Hoernig, Anne M. Brown, Christopher B. Lawrence, Mohamed N. Seleem

**Affiliations:** 1 Department of Biomedical Sciences and Pathobiology, Virginia-Maryland College of Veterinary Medicine, Virginia Polytechnic Institute and State University, Blacksburg, Virginia, United States of America; 2 Center for One Health Research, Virginia Polytechnic Institute and State University, Blacksburg, Virginia, United States of America; 3 Department of Biomedical Sciences, Edward Via College of Osteopathic Medicine (VCOM), Blacksburg, Virginia, United States of America; 4 Purdue Institute for Cancer Research, Purdue University, West Lafayette, Indiana, United States of America; 5 Department of Medicinal Chemistry and Molecular Pharmacology, Purdue University, West Lafayette, Indiana, United States of America; 6 Department of Biochemistry, Virginia Polytechnic Institute and State University (Virginia Tech), Blacksburg, Virginia, United States of America; 7 Center for Emerging, Zoonotic, and Arthropod-borne Pathogens, Virginia Polytechnic Institute and State University (Virginia Tech), Blacksburg, Virginia, United States of America; 8 Research & Informatics and Department of Biochemistry, Virginia Polytechnic Institute and State University (Virginia Tech), Blacksburg, Virginia, United States of America; University of Florida, UNITED STATES OF AMERICA

## Abstract

Cryptococcosis is an opportunistic fungal infection affecting individuals with compromised immunity, particularly those with HIV. The limited accessibility to effective treatments and treatment-related toxicities underline the need for more effective therapeutic options. In this study, we conducted a whole-cell screening of ~ 3,700 FDA-approved drugs and clinical molecules against the *Cryptococcus neoformans* H99 strain. The anti-mycobacterial agent SQ109 was identified as one of the most potent hits, with broad antifungal activity. SQ109 exhibited potent activity against *Cryptococcus* spp*.*, with an MIC90 of 4 μg/mL. In the time-kill assay, SQ109 demonstrated a fungicidal activity on proliferating cryptococcal cells in a concentration-dependent manner. Unlike fluconazole (FLC) and flucytosine (5-FC), *C. neoformans* showed a negligible tendency to develop resistance to SQ109 during frequent passaging. Furthermore, SQ109 exhibited a potent efficiency in the murine model of cryptococcal infection, resulting in a 50% survival rate among animals treated with 25 mg/kg for 10 consecutive days. The transcriptomic analysis revealed that SQ109 disrupts ergosterol biosynthesis, affecting membrane integrity and oxidative homeostasis. Additionally, molecular docking and structural analysis indicated that squalene synthase protein ERG9 is the most likely target of SQ109 within the ergosterol biosynthesis machinery of cryptococcal cells. Notably, SQ109 potentiates the activity of the standard antifungal FLC, as well as other ergosterol inhibitors, with a fractional inhibitory concentration Index (ΣFICI) ranging from 0.38 to 1. These findings highlight the therapeutic potential of SQ109 in combating cryptococcal infections, both as a standalone therapy and as an adjuvant to FLC monotherapy.

## Author summary

In this study, we are investigating the antifungal attributes of an oral anti-mycobacterial agent (SQ109) against the *Cryptococcus neoformans/gattii* pathogen. This fungal pathogen contributes to pulmonary and cerebral infections among immunocompromised patients, with a mortality rate exceeding 60%. The limited treatment options, along with their availability and potential clinical complications, especially in resource-limited regions, underscore the need for more efficient treatment. SQ109 demonstrates potent fungicidal activity against cryptococcal cells. During frequent exposure, cryptococcal cells exhibit a negligible tendency to evolve resistance against SQ109, a crucial advantage given the extended treatment regimen for cryptococcal infection. SQ109 also demonstrates enhanced activity in combination with oral fluconazole, which could improve the therapeutic outcome of this infection in regions that rely on fluconazole monotherapy. The activity of SQ109 in the mouse model of cryptococcal meningitis further highlights the therapeutic potential of this drug in combating cryptococcal infection. Additionally, this study investigates potential targets and cellular responses to SQ109 within cryptococcal cells using varied molecular techniques and biological assays.

## Introduction

Cryptococcosis is a global health threat that primarily affects individuals with a weakened immune system [1]. This opportunistic infection is predominantly caused by inhaling fungal cells of *Cryptococcus neoformans* and, to a lesser extent, *C. gattii*. Invading fungal cells efficiently colonize the alveolar spaces and disseminate to the central nervous system, resulting in life-threatening cryptococcal meningitis (CM) [2,3]. Annually, cryptococcal infections account for approximately 152,000 new cases worldwide, with a mortality rate exceeding 60%, primarily attributed to limited access to proper diagnosis and effective treatment [4].

The current treatment regimen is limited to three systemic antifungals, including amphotericin B (AmB), flucytosine (5-FC), and fluconazole (FLC). Intravenous deoxycholate AmB (0.7-1 mg/kg/day) is typically combined with oral 5-FC (100 mg/kg/day) during the induction stage of treatment to provoke early fungal clearance [5,6]. Due to its fungistatic activity, oral FLC is used in moderate to low doses during the consolidation and maintenance stages [6]. The limited access to the AmB regimen, especially in heavily burdened countries, urges the use of FLC as an alternative monotherapy during the induction stage [7]. Unfortunately, this therapeutic alternative has inadequate outcomes, resulting in treatment failure and relapses in approximately 50% of treated individuals [8–10]. Therefore, identifying novel drugs remains a pressing need to leverage the therapeutic outcome for this infection. Approaching novel molecules acting on pathogen-specific targets presents a promising strategy to combat existing challenges related to emerging resistance and host toxicity [11]. However, repositioning existing drugs, with established pharmacokinetics and safety profiles, offers a more cost and time-efficient approach to replenish the therapeutic pipeline [12,13].

In this study, we screened 3,700 FDA-approved drugs and molecules being evaluated in clinical trials against *C. neoformans*. We identified SQ109 as one of the most potent hits, exhibiting fungicidal activity against *Cryptococcus* isolates with a minimum inhibitory concentration (MIC_90_) of 4 μg/mL. The antifungal activity of SQ109 against *Cryptococcus* yeast has also been reported in a recent study, along with a wide variety of filamentous and dimorphic fungal species [14]. This oral antimycobacterial agent has completed Phase 1 and Phase 2 clinical trials for the treatment of tuberculosis, and it has received Fast Track designation and orphan drug status from the U.S. Food and Drug Administration (FDA) [15,16].

The diverse pharmacokinetic properties of SQ109 support its therapeutic potential against cryptococcal infections. Its extended half-life, low plasma protein binding, and large volume of distribution favor drug accumulation in alveolar compartments at therapeutic levels exceeding MIC_90_ for *Cryptococcus* pathogens with frequent dosing [17–20]. Moreover, the lipophilic nature of SQ109, coupled with its relatively small molecular weight, enables efficient penetration of the blood-brain barrier, highlighting its potential role in combating the cerebral progression of cryptococcal infection [18,21]. Additionally, its superior safety profile during frequent dosing is typically crucial for the extended management of cryptococcal infection [15,22,23].

The transcriptomic analysis of treated cryptococcal cells in response to SQ109, in addition to the increased susceptibility of heterozygous ergosterol mutants of *C. albicans*, clearly reveals its impact on ergosterol biosynthesis. Squalene synthase protein (ERG9) was identified as a potential target for SQ109 within this pathway. The negligible tendency of cryptococcal cells to develop resistance to SQ109, combined with its compelling efficacy in the survival murine model of CM and its synergistic-to-additive interaction with FLC, highlights the therapeutic potential of SQ109 in treating cryptococcal infections.

## Results

### Identification and evaluation of SQ109 antifungal activity against *Cryptococcus neoformans*

**Fig 1:**
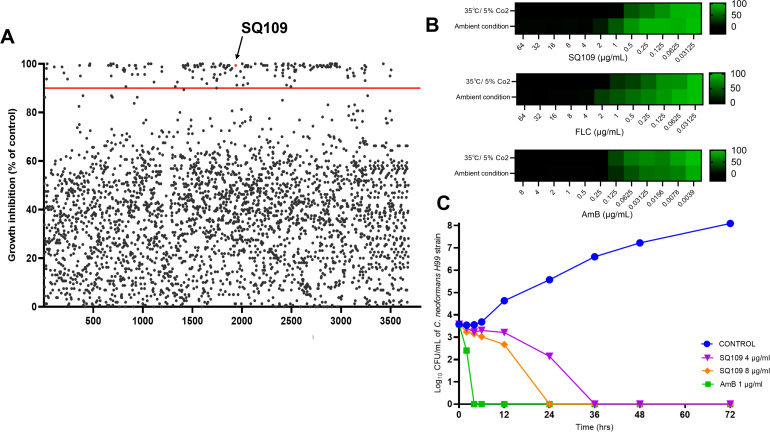
Identification and evaluation of the SQ109 activity on the *Cryptococcus* fungal pathogen. **(A)** Screening of MCE drug repurposing library against *C. neoformans* H99 strain. Individual compounds (~3700) were screened at a concentration of 16 μM in RPMI-MOPS medium. The optical density (OD) of treated H99 cultures was measured at 540 nm after 72 hrs. All compounds are represented by black dots, indicating their varying inhibitory activities. SQ109 (red dot) was identified as one of the most potent hits, inhibiting fungal growth above the cut-off level (solid red line). **(B)** Susceptibility of *C. neoformans* H99 strain to SQ109 and standard antifungals, FLC and AmB, under ambient and host-like levels of CO_2._ OD was measured at 540 nm and was presented as heat maps relative to the growth of the untreated culture. **(C)** Growth kinetics of *C. neoformans* H99 inocula (~5 x10^3^ CFU/mL) individually treated with SQ109 (4 and 8 µg/mL) and AmB (1 µg/mL), as a control antifungal, over 72 hrs in RPMI-MOPS medium. Aliquots of treated cultures were serially diluted (1:10) and plated on YPD agar at different time points (0, 2, 4, 8, 12, 24, 36, 48, and 72 hrs) to monitor the effect of SQ109 on the proliferation of treated cells.

We conducted a whole-cell screening of ~3,700 therapeutic entities, including FDA-approved drugs and molecules from clinical trials, against the *C. neoformans* H99 strain. Molecules were screened at a fixed concentration of 16 μM, and SQ109 was identified among the most potent hits, achieving ~100% inhibition of fungal growth (Fig 1A, S1 Data).

The antifungal activity of SQ109 was validated on a panel of 18 clinical isolates representing the two major pathogenic species of *Cryptococcus* (*C. neoformans* and *C. gattii*). Additionally, clinical isolates of other fungal pathogens, *Aspergillus fumigatus* and multidrug-resistant *Candida* species, were included in the susceptibility study. SQ109 exhibited broad-spectrum antifungal activity against the tested fungal species, with a remarkable potency against the *C. neoformans/gattii* complex, achieving its inhibitory activity at an MIC_90_ of 4 μg/mL (Table 1). Additionally, we evaluated the activity of SQ109 at an increased level of CO_2_ (~5%), mimicking the host conditions within the lung. Like FLC, SQ109 exhibited an increased activity against cryptococcal cells at 5% CO_2_, with a 2-fold reduction in its MIC compared to ambient conditions. On the other hand, AmB exhibited consistent activity against cryptococcal cells under both conditions, with no change in its MIC (Fig 1B). The impact of SQ109 on the growth kinetics of cryptococcal cells was monitored at various time points over 72 hours. Notably, SQ109 exhibited a concentration-dependent fungicidal effect on proliferating cryptococcal cells (Fig 1C). Similarly, SQ109 demonstrated potent fungicidal activity against quiescent cryptococcal cells, clearing the entire treated inoculum within 2 hours (Fig A in S1 Text).

**Table 1:** Antifungal activity of SQ109 on clinical isolates of *Cryptococcus* spp*.* and other fungal pathogens.

Isolate	MIC (µg/mL)		
	SQ109	FLC	AmB
*C. neoformans* H99 ATCC208821	2	4	0.25
*C. neoformans* NR-41291	4	4	0.5
*C. neoformans* NR-41292	4	8	1
*C. neoformans* NR-41295	4	16	0.5
*C. neoformans* NR-41296	4	4	1
*C. neoformans* NR-41297	4	2	0.5
*C. neoformans* NR-41298	4	4	1
*C. neoformans* NR-41299	4	4	1
*C. neoformans* NR-41300	4	1	1
*C. neoformans* NR-50333	2	1	0.5
*C. gattii* R265 NR-43208	4	4	1
*C. gattii* CBS1930 NR-43209	4	8	1
*C. gattii* NR-50422	4	16	1
*C. gattii* NR-50423	4	8	1
*C. gattii* NR-50425	4	8	1
*C. gattii* NR-50426	2	4	1
*C. gattii* NR-50427	2	4	0.5
*C. gattii* NR-50429	4	2	0.5
*A. fumigatus* AR0736	32	>128	1
A. *fumigatus* AR0738	16	>128	1
*C. albicans* SC5314	32	0.5	1
*C. auris* AR0388	8	>128	2
*C. auris* AR0390	8	>128	2
*C. parapsilosis* ATCC 22019	16	1	1
*C. tropicalis* ATCC 13803	8	2	1

### RNA-sequencing of SQ109-treated cryptococcal cells

**Fig 2:**
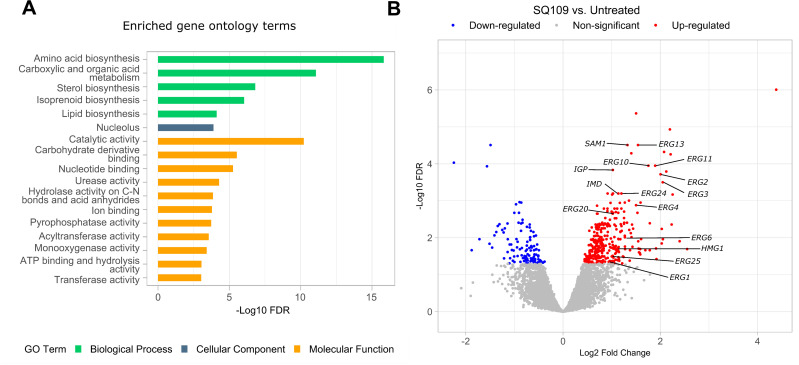
Transcriptomic and Gene Ontology (GO) analysis of SQ109-treated *C. neoformans* H99 cells. **(A)** GO analysis of the impacted biological processes, cellular components, and molecular function following brief exposure to SQ109 (2 μg/mL). Metabolic processes involved in the biosynthesis of different molecules, including amino/carboxylic acids, isoprenoid intermediates, and sterols were significantly upregulated (FDR ≤ 0.05) in response to SQ109 treatment. **(B)** The volcano plot highlights 501 differentially expressed genes (DEGs) with an FDR of ≤ 0.05.

To identify cellular pathways affected by SQ109 treatment, we conducted a transcriptomic analysis of *C. neoformans* H99 following a brief exposure to SQ109 (2 μg/mL). The transcriptomic profile of treated cells was compared to the control (DMSO-treated) cells. Five hundred and one (501) genes exhibited a significant expression change (FDR < 0.05). Gene ontology (GO) analysis highlighted various impacted molecular functions involved in the biosynthesis of essential macromolecules, including carboxylic acids, isoprenoid intermediates, and sterols (Fig 2A). Notably, 12 genes involved in the ergosterol biosynthesis including *HMG1, ERG2, ERG3, ERG4, ERG6, ERG9, ERG10, ERG11, ERG13, ERG20, ERG24,* and *ERG25* exhibited a substantial enrichment (~2-4.3 fold) in response to SQ109 treatment (Fig 2B).

### Impact of ergosterol biosynthesis perturbation on the activity of SQ109

**Fig 3:**
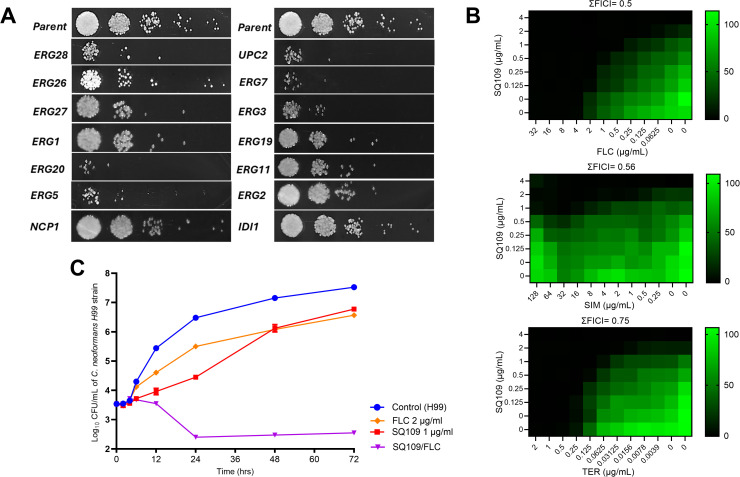
Validating the effect of SQ109 on the ergosterol biosynthesis machinery. **(A)** Impact of sublethal concentration of SQ109 (8 µg/mL) on a collection of heterozygous ergosterol mutants of *C. albicans* SC5314. Yeast inocula were adjusted to a final density of ~ 1 × 103 CFU/mL in RPMI-MOPS medium and incubated at 35 °C for 24 hrs in the presence of SQ109. Aliquots of treated cultures were diluted and spotted on YPD agar to evaluate cell depletion. **(B)** Interaction between SQ109 and different ergosterol inhibitors, including fluconazole (FLC), simvastatin (SMV), and terbinafine (TER), using the checkerboard microdilution technique on the *C. neoformans* H99 strain. **(C)** Time-kill assay demonstrating the enhanced killing activity of SQ109 (1 μg/mL) combined with FLC (2 μg/mL) on the proliferation of the *C. neoformans* H99 strain.

To validate the impact of SQ109 on ergosterol biosynthesis, we investigated whether the depletion of ergosterol biosynthetic machinery could affect susceptibility to SQ109. Therefore, we performed a fitness profiling utilizing a collection of heterozygous *C. albicans SC5314* deletion (haploinsufficient) mutants, representing 14 disrupted genetic loci (*IDI1, ERG19, ERG20, ERG26, ERG28, ERG27, ERG1, ERG5, NCP1, UPC2, ERG7, ERG3, ERG11, and ERG2*) involved in the isoprenoid/mevalonate pathway and the late part of the ergosterol pathway. Individual mutants were treated with a sublethal concentration of SQ109 (8 µg/mL) in RPMI-MOPS medium for 24 hrs to identify depleted mutants. As anticipated, most tested mutants exhibited significant growth inhibition (- Log10 ≥ 2) compared to the parent strain (Fig 3A), indicating increased susceptibility to SQ109.

### Interaction between SQ109 and other inhibitors for ergosterol biosynthesis

We tested the interaction between SQ109 and other ergosterol pathway inhibitors, including standard FLC targeting ERG11, terbinafine (TRB) targeting ERG1, and simvastatin (SMV) targeting HMG1 enzyme. Combining SQ109 with these inhibitors demonstrated enhanced activity against *C. neoformans* H99 strain at sublethal levels (Fig 3B). The interaction between FLC and SQ109 was further evaluated on the entire panel of *Cryptococcus neoformans/gattii* isolates, included in the susceptibility testing, using checkerboard microdilution. Remarkably, SQ109 exhibited either synergistic or additive interactions with FLC among 61% and 39% of the tested isolates, respectively, with ΣFICI ranging from 0.38 to 1 (Table A in S1 Text). This combination exhibited enhanced killing activity, reducing the proliferated fungal population by more than 3 log_10_ CFU/mL within 48 hrs (Fig 3C).

### Impact of SQ109 on membrane integrity and cellular stress response

**Fig 4:**
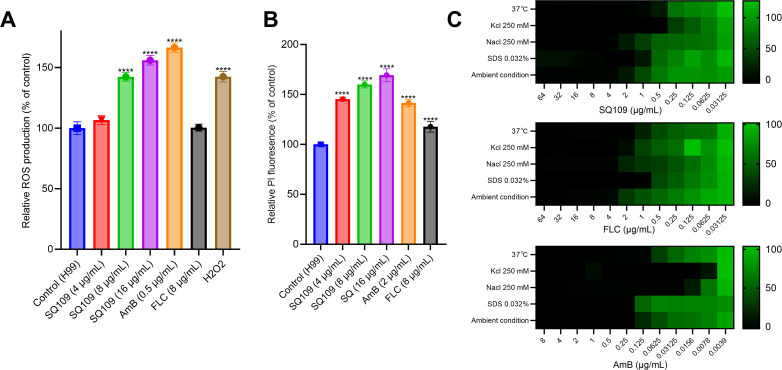
Impact of the SQ109 on the oxidative homeostasis and membrane integrity of treated cryptococcal cells. **(A)** ROS measurement within SQ109-treated cryptococcal cells using 2′,7′-dichlorodihydrofluorescein diacetate (H_2_DCFDA) stain at a concentration of 20 µM alongside hydrogen peroxide (H_2_O_2_) as a positive control and standard antifungals, AmB and FLC. **(B)** Membrane integrity of cryptococcal cells treated with either SQ109 or standard antifungals, AmB and FLC, using propidium iodide (PI) fluorescent probe at a concentration of 10 µM. **(C)** Impact of different cell stressors on the activity of SQ109. The susceptibility of *C. neoformans* H99 strain to SQ109 alongside standard antifungals at increased temperature (37 °C) and in the presence of 0.032% of sodium dodecyl sulphate (SDS), NaCl, and KCL (250 mM) in RPMI-MOPS medium. OD was measured at 540 nm, and relative growth intensities were presented as heat maps. Statistical significance was determined using a one-way analysis of variance test (ANOVA), where **** indicates P value < 0.0001.

Due to the impact of SQ109 on the ergosterol biosynthetic machinery, we investigated its effect on oxidative homeostasis and membrane integrity using 2′,7′-dichlorodihydrofluorescein diacetate (H_2_DCFDA) and propidium iodide (PI) fluorescent probes. Treatment with SQ109 at varying concentrations resulted in a significant, dose-dependent increase in the fluorescence of both indicators in treated cells (Fig 4A and 4B). Additionally, we evaluated the susceptibility of cryptococcal cells to SQ109 in the presence of various cell stressors that affect membrane fluidity, including sodium dodecyl sulfate (SDS), osmotic stressors (NaCl and KCl), and elevated temperature (37 °C). Like FLC, SQ109 displayed an enhanced activity against treated cryptococcal cells in the presence of all stressors, supporting its effect on membrane integrity. On the other hand, osmotic stressors and incubation at 37 °C augmented the activity of AmB, while SDS didn’t exert any change on its MIC (Fig 4C).

### Molecular docking of SQ109 in ergosterol biosynthesis targets

RNA and haploinsufficiency mutant studies highlighted several potential targets for SQ109 within the ergosterol biosynthesis pathway, with particular emphasis on the gene products of *ERG1*, *ERG11*, and *ERG9*, encoding squalene epoxidase, lanosterol-14-demethylase, and squalene synthase, respectively. Notably, the protein product of *ERG9* (squalene synthase) was reported to be a potential target for SQ109 in *T. cruzi* [24,25], which served as the basis for ERG9 target selection. Molecular modeling and docking, along with quantitative and qualitative analysis, were employed to predict the binding mode of SQ109 in each structure and to identify the most likely target.

Since no crystal structures are avaliable for any of the three proteins studied in *C. neoformans*, AlphaFold3 [26] was used to generate a valid structure for each (Fig C-E in S1 Text). Redocking was performed in homologous crystal structures from *H. sapiens, T. cruzi, and S. cerevisiae* for each protein (PBD IDs: 6C6P, 3WSB, and 4LXJ) [27], and SQ109 was docked into each model using GNINA [28]. Predicted free energy of binding was calculated, and the pose with the lowest molecular mechanics with generalized born and surface area solvation (MM/GBSA) was chosen for further analysis. The average predicted free energy of binding of all poses docked into *C. neoformans* ERG1 was less favorable than both ERG9 and ERG11 (214.5 kcal/mol versus –17.6 and –36.0 kcal/mol), indicating a less favorable interaction pattern and binding mode than in other structures and a less likely target of SQ109 (Fig. H in S1 Text).

**Fig 5:**
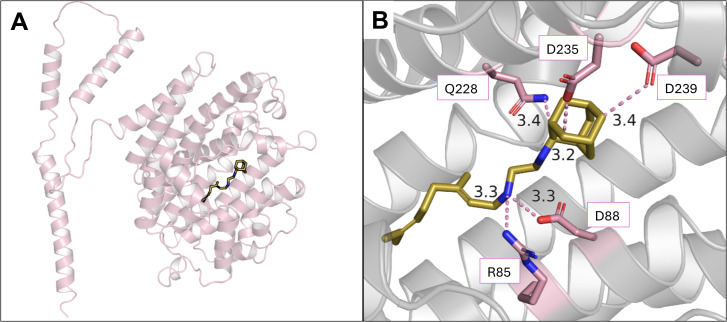
Molecular docking of SQ109 into the AlphaFold3-predicted squalene synthase (ERG9) protein of *C. neoformans.* **(A)** ERG9 protein is shown as a pink cartoon with the top pose of docked SQ109 (yellow sticks) occupying the binding site. **(B)** Key interactions (polar, Van Der Waals, and hydrogen) of docked SQ109 (yellow sticks) in the *C. neoformans* ERG9 protein (grey cartoon) with interacting amino acid sidechains (pink sticks). Interactions are highlighted with pink dashed lines and labelled with distance in Angstroms.

Interaction fingerprints were obtained to predict protein-ligand interactions (hydrophobic, hydrogen bonding, electrostatic, and aromatic) within a 3.5 Å radius. Of the three structures assessed, ERG11 showed no interactions within the cut-off range. The complete lack of interactions indicates that the binding site likely has little to no energetically favorable capacity to bind SQ109 within the interaction range, thus eliminating ERG11 as the most likely target. As expected, ERG9 showed both a favorable predicted free energy of binding and a variety of protein-ligand interactions (Fig 5A and 5B) within an acceptable range of 3.5 Å. These results, in addition to the conservation of key sequential and structural aspects between homologous ERG9 from *C. neoformans* and *T. cruzi,* with a global 38.95% amino acid sequential identity [29], and global alignment root-mean-square deviation (RMSD) of 0.905, suggesting that squalene synthase is the most likely target of SQ109 in *C. neoformans*. Additionally, other essential proteins within the ergosterol biosynthesis machinery (ERG19, ERG20, ERG26, ERG27, and NCP1) exhibited less favorable binding and fewer contact points with SQ109 compared to ERG9 (Fig I in S1 Text).

### Resistance acquisition study

**Fig 6:**
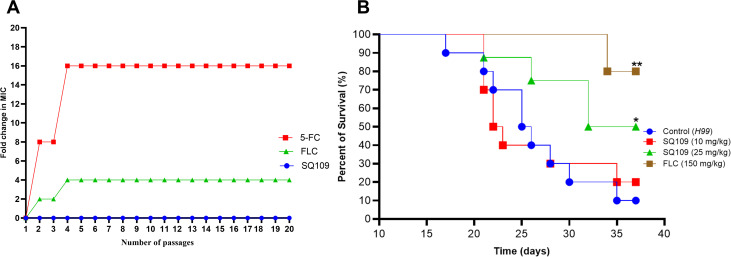
Evaluation of resistance tendency and the *in vivo* efficacy of SQ109 in the survival murine model of cryptococcal meningitis. **(A)** Resistance acquisition analysis of *C. neoformans* H99 cultures (~1 x 10^5^ CFU/mL) individually treated with sublethal concentrations (0.5 MIC) of either SQ109, fluconazole (FLC), or flucytosine (5-FC) in RPMI-MOPS medium for 20 passages. **(B)** Survival outcome of SQ109 in the murine model of CM. Immunocompetent C57BL/6 mice (10 per group) were intranasally infected with *C. neoformans* H99 (~1 × 10^6^ CFU). Mice were treated with SQ109 (10 and 25 mg/kg/day), standard FLC (150 mg/kg/day), or vehicle for 10 consecutive days. All treatments were given through oral gavage. Mice were monitored for 38 days for any signs of morbidity. Statistical significance was evaluated using log-rank (Mantel-Cox) analysis, where * and ** denote P value at *≤* 0.05 and 0.001, respectively.

Due to the heteroresistance nature of *Cryptococcus*, we evaluated whether cryptococcal cells could adapt to the activity of SQ109 during continuous treatment with a sublethal concentration. Individual cultures of *C. neoformans* H99 were treated with SQ109 and standard antifungals, including FLC and 5-FC, using a sublethal concentration (0.5 MIC) over 20 passages. Remarkably, cryptococcal cells had a negligible tendency to develop resistance to SQ109. In contrast, treated cryptococcal cells displayed a rapid adaptation to the antifungal activity of FLC and 5-FC, resulting in a 4- and 16-fold increase in the MIC, respectively, within the first 4 passages (Fig 6A).

### *In vivo* efficacy of SQ109 in the murine model of cryptococcal meningitis

To validate the therapeutic potential of SQ109, we investigated whether it could combat the progression of cryptococcal infection in a murine model of CM. Immunocompetent C57BL/6 mice were intranasally infected with *C. neoformans* H99 strain. Oral treatment with two different doses of SQ109 was initiated 24 hours post-infection for 10 consecutive days. Ninety percent of the untreated mice succumbed to the infection within 38 days. In contrast, mice treated with SQ109 at a dose of 25 mg/kg demonstrated a statistically higher survival rate (50%) compared to the control group and those treated with 10 mg/kg (Fig 6B).

## Discussion

Cryptococcal infection is a top-ranked invasive fungal infection that predominantly affects individuals with compromised immune systems [30,31]. The clinical manifestation of this infection depends on the intensity of the immunosuppression, with fatal consequences in severely immunocompromised individuals [32]. Historically, cryptococcal infection has been linked to HIV infection, accounting for 19% of HIV-related mortality [4]. However, immune dysregulation secondary to the extensive use of immunosuppressive therapies and emerging viral and bacterial infections has heightened the risk of cryptococcal infections among non-HIV population [33–36].

While drug repurposing offers a more efficient approach for identifying therapeutic alternatives, addressing the varied physicochemical and pharmacokinetic requirements to combat such invasive infections poses a significant challenge, often necessitating structural modification [37,38]. In this study, we identified the 1, 2-ethylenediamine compound (SQ109) as one of the most potent hits, obtained from screening ~ 3,700 therapeutic entities, against the *C. neoformans* H99 strain. Notably, SQ109 exhibited broad-spectrum antifungal activity, being more potent against *Cryptococcus* isolates with a MIC_90_ of 4 μg/mL. Inside the lungs, cryptococcal cells are adapting to the increased level of CO2 (~5%), which has been reported to modulate the activity of standard antifungals [39,40]. Interestingly, the activity of SQ109 was enhanced at the host level of CO2, similar to FLC and 5-FC, highlighting its potential activity against the colonizing cryptococcal cells within lung compartments. In the time-kill assay, SQ109 demonstrated potent fungicidal activity against both proliferating and quiescent fungal cells, which is highly desirable for treating CM, given the fungistatic nature of FLC monotherapy.

SQ109 primarily targets the MmpL3 transporter within *Mycobacterium tuberculosis*, thereby disabling the incorporation of mycolic acid into the growing cell wall [41]. Interestingly, no conserved homolog for this transporter has been identified within other pathogens, suggesting the presence of an unidentified molecular target within fungi [42]. Furthermore, SQ109 was previously reported to impact the mitochondrial function and calcium homeostasis in trypanosomatid parasite and *Saccharomyces cerevisiae,* which indicates the multi-targeting nature of SQ109 [14,24]. Therefore, we conducted a transcriptomic analysis for cryptococcal cells to elucidate potential cellular responses to SQ109. The statistically significant gene expression, coupled with ontology analysis, highlighted the impact of SQ109 on genes involved in the biosynthesis of isoprenoid and sterol intermediates essential for ergosterol production. This transcriptomic response exhibits a remarkable similarity to that elicited under FLC exposure, characterized by a substantial upregulation of *ERG11*, which encodes lanosterol 14-alpha-demethylase, the target of FLC, as well as other genes within the late biosynthetic pathway, including *ERG1, ERG2, ERG3,* and *ERG25*. Additionally, *ERG13* encoding the production of hydroxymethylglutaryl-CoA synthase within the isoprenoid pathway exhibited a similar upregulation in response to SQ109 and FLC treatment [43,44].

Ergosterol is the main component of the fungal cell membrane, maintaining its fluidity and integrity [45]. The biosynthetic machinery of this sterol comprises 25 proteins controlling its production within the endoplasmic reticulum. Disrupting these enzymatic loci halts ergosterol supply and perturbs the integrity of the cell membrane [44,46]. Consequently, the accumulation of toxic sterol intermediates triggers oxidative stress within the fungal cells, ultimately contributing to cellular death [47,48]. In our study, SQ109 significantly elevated the accumulation of reactive oxygen species (ROS) within cryptococcal cells. Additionally, treated fungal cells exhibited a significant loss of membrane integrity in response to varying concentrations of SQ109. These cellular changes are consistent with the effect of FLC and other repurposed ergosterol inhibitors on several yeast pathogens [49,50].

To validate the impact of SQ109 on ergosterol biosynthesis, we evaluated the ability of *C. albicans* SC5314 mutants with heterozygous deletions at different enzymatic points within the ergosterol pathway to tolerate SQ109 at a sublethal concentration. Most of the tested mutants exhibited haploinsufficiency in response to the used concentration. Strains with defective loci within the late ergosterol pathway (*ERG28, ERG26, ERG27, ERG5, ERG7, and ERG3*) and the isoprenoid/mevalonate pathway (*ERG19* and *ERG20*) were significantly depleted (-Log10 ≥ 2). Interestingly, mutants with individual deletions in the two key enzymatic points, *ERG11* and *ERG1*, encoding squalene epoxidase, exhibited reduced tolerance to SQ109. Furthermore, *Candida* strain with a mutation in the *UPC2* transcription factor, controlling sterol uptake and the activity of the ergosterol biosynthetic genes, was significantly depleted, aligning with other studies revealing the increased susceptibility of *UPC2* mutants to azoles and other ergosterol inhibitors [51–54]. Consistently, pharmacological manipulation of the ergosterol pathway using different allosteric inhibitors, including SIM, TER, and FLC, showed enhanced activity when combined with a sublethal concentration of SQ109 against cryptococcal cells. This beneficial interaction was also reported between SQ109 and pitavastatin against *Saccharomyces cerevisiae* [14]*,* validating its impact on ergosterol biosynthesis among fungal pathogens.

Molecular docking of SQ109 within potential target proteins highlighted its favorable binding to the squalene synthase (ERG9) protein of cryptococcal cells. This result aligns with the ability of SQ109 to target the ERG9 protein in *T. cruzi* [24]. Since SQ109 is less active against *A. fumigatus,* molecular modeling was employed to explore the binding modes of SQ109 in homologous ERG9 proteins from *A. flavus, T. cruzi,* and *C. neoformans,* and to assess the sequential and structural differences between these homologous proteins. The available structure of *T. cruzi* [24] was co-crystallized with SQ109 and serves as the basis for redocking verification via RMSD. The ERG9 structure from *A. flavus* [55] was utilized for molecular docking and was compared to the homologous structures from *C. neoformans* and *T. cruzi*.

Comparative structural analysis of ERG9 from *A. flavus*, *T. cruzi*, and *C. neoformans* reveals conservation of key sequences in the active site responsible for squalene synthesis, namely **Y**XXYCYYVAGLVGXGLXXL and FX**F**SAIPQXMAXXTL. These contain a tyrosine and phenylalanine residue, respectively, which catalyze the first and second half-reactions of squalene synthesis. These key residues exhibit the same orientation within the binding pocket in all structures assessed, making it unlikely that there is an active site difference that would influence the binding of SQ109. Additionally, the docking results of SQ109 into *A. flavus* ERG9 protein positioned SQ109 in the active site with several interacting residues (F55, I59, Y74, L77, D81, V184, A185, G189) and a favorable average predicted free energy of binding between poses (-43.3 kcal/mol average, -50.5 kcal/mol best pose) (Fig H in S1 Text). This suggests that the reduced antifungal activity of SQ109 in *A. fumigatus* may be related to other factors that could limit access to the binding cavity of the active site.

A notable difference between the *A. flavus* structure and others assessed is a motif of repeat aspartate residues at the binding site entrance (DDDR), which is not observed in *T. cruzi* (VPPR) or *C. neoformans* (IDGR). This motif is one of two conserved repeat sequences that flank the entrance to the binding site, oriented across the active site from a pair of aspartate residues. Of these, only one is conserved across all structures and coordinates a magnesium ion during catalysis, while the second, unique to *A. flavus*, may obstruct SQ109 binding and contribute to its reduced efficacy [56]. It is hypothesized that flanking aspartate residues in the binding site entrance of *A. flavus* squalene synthase prevent the large, nonpolar adamantane head of SQ109 from entering the active site, while still allowing farnesyl diphosphate head access due to the higher polarity of the diphosphate head. Binding pocket visualizations using CavitOmiX (v. 1.0, 2022, Innophore GmbH) show that of the three structures, the *A. flavus* squalene synthase is more polar at the binding site entrance than the homologous structures (Fig. G in S1Text). In homologous structures of *T. cruzi* and *C. neoformans*, the motif primarily comprises smaller, nonpolar side chains that are less likely to hinder the adamantane head of SQ109 (Fig. G in S1 Text).

Mutation frequency and the intrinsic heterogeneity of *Cryptococcus* represent another challenge for repurposing SQ109. The ability of cryptococcal cells to tolerate higher concentrations under azole stress, secondary to their intrinsic heterogeneity, mainly contributes to relapses and treatment failure with FLC monotherapy [57]. Similarly, 5-FC rapidly induces stable mutation in treated cryptococcal cells, limiting its role as a monotherapy [58]. Notably, *Cryptococcus* displays a negligible propensity to overwhelm the activity of SQ109 during repeated exposure, highlighting the potential therapeutic advantage of SQ109 over FLC monotherapy. This negligible propensity is consistent with the reported activity of SQ109 against FLC-resistant fungal species [14], which also highlights ERG9 protein as a potential target for SQ109 within the ergosterol pathway. The observed synergistic-to-additive interaction between SQ109 and FLC also draws attention to its potential therapeutic role as an adjuvant to FLC, aiming to enhance the effectiveness of the current oral treatment regimen. Furthermore, the survival benefit of SQ109 in the murine model of disseminated CM, at a therapeutically achievable concentration, validates its therapeutic potential in managing cryptococcal infections.

Overall, this study elucidates the diverse in vitro attributes of SQ109 as a promising therapeutic candidate against the *Cryptococcus* pathogen. The pharmacokinetic traits and *in vivo* efficacy of SQ109 support its potential as an oral treatment, either as a monotherapy or in combination with FLC, against this invasive infection.

## Materials and methods

### Ethical approval

The Virginia Tech Institutional Animal Care and Use Committee has approved the animal experiment included in this study [IACUC number: 23–185 (CVM)].

### Fungal isolates, media, chemicals, and tested drugs

A total of 18 clinical isolates representing *C. neoformans/gattii* complex were included in this study. In addition, 6 clinical isolates of clinically relevant fungi, including *Candida parapsilosis*, *C. tropicalis, C. auris*, and *Aspergillus fumigatus* were involved in susceptibility testing. Fungal strains were obtained from the American Type Culture Collection, ATCC (Manassas, VA, USA), BEI Resources (Manassas, VA, USA), and the US Centers for Disease Control and Prevention, CDC (Atlanta, GA, USA). Culturing media, chemicals, and drugs used in this study were obtained from the following vendors: RPMI 1640 (Gibco, NY, USA), 3-(N-Morpholino) propane sulfonic acid (Fisher Bioreagents, NJ, USA), yeast potato dextrose (YPD) agar and broth (Becton, Dickinson and Company, MD, USA), sodium chloride, potassium chloride, 30% hydrogen peroxide solution, and sodium dodecyl sulphate (Sigma-Aldrich, MO, USA), propidium iodide (Chem-Impex International, IL, USA), 2′,7′-dichlorodihydrofluorescein diacetate (Tocris Bioscience, MN, USA), SQ109 (MedKoo Biosciences, NC, USA), AmB (Chem-Impex International, IL, USA), FLC (Thermo Fisher, NC, USA), 5-FC (Thermo Scientific, MA, USA) terbinafine hydrochloride (Sigma-Aldrich, MO, USA), simvastatin (TCI, PA, USA).

### Drug library screening and the identification of SQ109 activity

Antifungal activity screening was conducted on the drug repurposing compound library (HY-L035) obtained from MedChem Express (MCE) against the *C. neoformans* H99 strain as previously described [59,60]. A total of 3,700 therapeutic compounds were screened at a fixed concentration of 16 µM in RPMI-MOPS medium, with the fungal inoculum standardized to approximately 1 × 103 CFU/mL. The optical density (OD) of each culture was measured at 540 nm, 72 hrs post-incubation at 35 °C, and expressed relative to control growth. Compounds with a growth inhibition of 90% or greater were classified as potential hits.

### In vitro drug susceptibility and microdilution checkerboard assay

Determination of the MIC for SQ109 and standard antifungals, AmB and FLC, was conducted as previously described in the Clinical and Laboratory Standards Institute (CLSI) guidelines M27-A3 for yeast and M38-A2 for filamentous fungi [61,62]. Colonies of tested *Cryptococcus* and *Candida* strains from YPD agar were resuspended in sterile potassium phosphate buffer (PBS) and adjusted to a final density of ~ 1 × 103 CFU/mL in RPMI-MOPS medium. Similarly, the inoculum for the included filamentous fungal strains was standardized to a final density of ~2 × 10^4^ CFU/mL in RPMI-MOPS. Susceptibility interpretation was conducted 72 hrs post-incubation at 35 °C for *Cryptococcus* strains and 24 hrs post-incubation for the tested *Candida* strains. The susceptibility of the tested filamentous fungi was evaluated 48 hours post-incubation at 35 °C. The in vitro interaction between SQ109 and FLC was performed using the standard microdilution checkerboard method as previously described [63,64]. The fractional inhibitory concentration index (ΣFICI) was calculated to interpret the interaction between the two drugs. FICI values of ≤ 0.5 or between 0.5 and 1 indicate synergistic and additive interaction, respectively, while FICI values between 1 and 2, or > 4 indicate indifferent and antagonistic interaction [65,66].

### Time kill assay

Evaluation of the SQ109 effect on the proliferation of cryptococcal cells was performed as previously described [60,63,65]. Exponentially growing *C. neoformans* H99 cells in YPD broth were used to inoculate the RPMI medium at a final density of ~ 5 x10^3^ CFU/mL. Individual cultures were treated with SQ109 at different concentrations and incubated at 35 °C. AmB (1 µg/mL) was involved as a positive control. Aliquots from treated cultures were diluted and spotted on YPD agar at specific time points (0, 2, 4, 6, 12, 24, 36, 48, and 72 hrs). Viable cells were enumerated after incubation at 35 °C for 48 hrs and plotted versus time points to establish the growth kinetic curve.

### Transcriptomic analysis of SQ109-treated cells

#### RNA extraction.

Exponential *C. neoformans* H99 cells from YPD culture were pelleted, washed three times with saline, and adjusted to a final density of ~1 x10^6^ CFU/mL in RPMI-MOPS medium. Individual aliquots were treated with SQ109 (2 μg/mL) or DMSO (control) for 3 hrs at 35 °C under agitation. Treated yeast cells were collected, and RNA extraction was performed using RiboPure-Yeast Kit (Ambion, AM1926, MA, USA) following manufacturer guidelines [64,67]. The quality of RNA samples was validated using NanoDrop One spectrophotometer (Thermo Fisher Scientific, DE, USA).

### Transcriptomic and enrichment analysis of differentially expressed genes

Illumina NovaSeq X Plus was used to sequence 150-bp paired-end DNA samples, and adaptor sequences were eliminated using Fastp [68]. Reads were aligned to the reference genome of *C. neoformans var. grubii* H99 GCF_000149245.1 using HISAT2 v2.0.5 [69,70]. Filtered and normalized gene expression levels were calculated from the aligned reads using HTSeq v.2.0.8 [71]. Differential gene expression was calculated by linear modeling and Bayesian statistics using the limma v.3.49.1 for R [72]. The Benjamini-Hochberg method was used to adjust P values for multiple testing. The cut-off value for significant differential expression was set at an FDR of 0.05.

### In silico methods for molecular docking and analysis

Given the lack of available crystal structure data of *C. neoformans* ERG1, ERG9, or ERG11, AlphaFold3 was utilized to predict a 3D structure of each protein [26]. Verification procedures were employed to assess the overall quality of each of the 5 output AlphaFold models, assessing for agreement between 3D and 1D structure and between the model and existing crystal structures [73–75]. The *T. cruzi* and *A. flavus* crystal squalene synthase (PDB IDs: 3WSB [24] 7WGH [55]), *H. sapiens* squalene epoxidase (PDB ID: 6C6P, chain A) [76], and *S. cerevisiae* lanosterol-14-demethylase (PDB ID: 4LXJ) [27] were selected for redocking. Redocking was performed using GNINA [28] with box coordinates specified in Table B inS1 Text and a box size of (30 x 30 x 30 Å) to confirm the ability to replicate resolved ligand placement in each binding pocket. Each redock yielded nine poses with best pose RMSD values outlined in Table B in S1 Text, indicating reasonable alignment of SQ109 docking poses to crystallographic data. The AlphaFold3 structures were aligned or superposed to their respective crystal structures to utilize the same workflows outlined in Table B in S1 Text. For docking, SQ109 was extracted from PDB ID: 3WSB as.pdb for import into GNINA and docked into the *A. flavus* squalene synthase and each *C. neoformans* AlphaFold3 structure. Each pose was evaluated using Schrodinger Maestro v2025-1. Visualizations were performed in PyMOL v. 3.0. From these poses, interaction fingerprints and predicted free energy of binding (MM/GBSA) were obtained, with interaction fingerprints identified by contacts between heavy atoms involved in the same type of interaction (e.g., hydrogen bonds, van der Waals interactions) within a distance threshold of 3.5 Å [77,78].

### Assessment of membrane integrity and oxidative stress

Aliquots of *C. neoformans* H99 inoculum, from an overnight culture in YPD broth, were adjusted to a final density of ~1x10^6^ CFU/mL in RPMI-MOPS medium and treated with SQ109 for 4 hrs at 35 °C. To detect the damage in the fungal membrane, treated cells were stained with 10 µM propidium iodide (PI) for 20 minutes. The resulting fluorescence was measured at an excitation wavelength of 488 nm and an emission wavelength of 617 nm using a BioTek Synergy H1 microplate reader [79]. Similarly, treated cells were stained with 2′,7′-dichlorodihydrofluorescein diacetate (H_2_DCFDA) at a final concentration of 20 µM to evaluate the accumulation of reactive oxygen species. The fluorescence signal was measured at an excitation wavelength of 485 nm and an emission wavelength of 530 nm [48].

### Analysis of resistance development

The ability of cryptococcal cells to adapt to the antifungal activity of SQ109 was evaluated using repeated passaging as previously described [49,80]. An inoculum of *C. neoformans* H99 (~1x10^5^ CFU/mL) was continuously exposed to 0.5 MIC of SQ109 in RPMI-MOPS medium and incubated under shaking conditions at 35 °C for 48 hrs. Standard antifungals, including FLC and 5-FC, were used as control antifungals. Treated cryptococcal cells were used to inoculate the next passage and to determine MIC following each passage. Frequent passaging was conducted for a total of 20 passages.

### Murine model of cryptococcal meningitis

Evaluation of the *in vivo* efficacy of SQ109 was conducted in the survival murine model of CM as previously described [81–84]. Immunocompetent C57BL/6 mice (6–8 weeks, 10 mice in each group) were anesthetized with isoflurane and intranasally infected with ~1x10^6^ CFU of *C. neoformans* H99 in 20 μL of saline. The inoculum was confirmed by 1:10 serial dilution in saline and plating on YPD agar. Treatment was initiated 24-hour post-infection and continued for ten consecutive days. Mice were treated with different doses of SQ109 (10 and 25 mg/kg/day) or vehicle through oral gavage. Fluconazole (150mg/kg/day) was used as a control antifungal. Mice were monitored throughout the experimental period for any sign of morbidity, including heavy breathing, abnormal gait, fur ruffling, and weight loss.

### Statistical analysis

All collected data were subjected to statistical analysis utilizing GraphPad Prism 8. The P values for multiple comparisons were calculated through a one-way analysis of variance (ANOVA). The statistical significance of survival probability in the animal experiment was evaluated using the log-rank (Mantel-Cox) test. P value of ≤ 0.05 indicated the statistical significance between the compared data sets.

## Supporting information

S1 Data Hits from the HY-L035 drug repurposing library. (XLSX)

S2 Data DEGs of SQ109-treated cryptococcal cells. (XLSX)

S1 Text *In vitro* activity and molecular docking supporting information. (DOCX)
