# Supplementary material for: In vitro and in vivo efficacy of the antimycobacterial molecule SQ109 against the human pathogenic fungus, Cryptococcus neoformans
Source: PLoS Negl Trop Dis. 2025 Dec 16;19(12):e0013837. doi: 10.1371/journal.pntd.0013837 (PMC12716712; doi:10.1371/journal.pntd.0013837)
Supplement: S1 Text — (DOCX) [file pntd.0013837.s003.docx]

**SUPPLEMENTARY MATERIAL**

***In vitro* and *in vivo* efficacy of the antimycobacterial molecule SQ109 against the human pathogenic fungus, *Cryptococcus* *neoformans***

Nour M. Alkashef ^a,b^, Ehab A. Salama ^a,b^, Ramu Anandakrishnan ^a,c^, Tony R. Hazbun ^d,e^, Micah V. Hoernig ^f^, Anne M. Brown ^f,g,h^, Christopher B. Lawrence ^a,b^, Mohamed N. Seleem ^a,b,#^.

^a^ Department of Biomedical Sciences and Pathobiology, Virginia-Maryland College of Veterinary Medicine, Virginia Polytechnic Institute and State University, Blacksburg, Virginia, 24061, USA.

^b^ Center for One Health Research, Virginia Polytechnic Institute and State University, Blacksburg, Virginia, 24061, USA.

^c^ Department of Biomedical Sciences, Edward Via College of Osteopathic Medicine (VCOM), Blacksburg, Virginia, USA

^d^ Purdue Institute for Cancer Research, Purdue University, West Lafayette, Indiana, USA.

^e^ Department of Medicinal Chemistry and Molecular Pharmacology, Purdue University, West Lafayette, IN, USA

^f^ Department of Biochemistry, Virginia Polytechnic Institute and State University (Virginia Tech), Blacksburg, VA, 24061, United States

^g^ Center for Emerging, Zoonotic, and Arthropod-borne Pathogens, Virginia Polytechnic Institute and State University (Virginia Tech), Blacksburg, VA, 24061, United States

^h^ Research & Informatics and Department of Biochemistry, Virginia Polytechnic Institute and State University (Virginia Tech), Blacksburg, VA, 24061, United States

^#^Corresponding Author:

Mohamed N. Seleem

Department of Biomedical Sciences and Pathobiology

Virginia-Maryland College of Veterinary Medicine

Virginia Polytechnic Institute and State University

1410 Prices Fork Rd, Blacksburg, Virginia, 24061

Phone: 540-231-7173

Email: [seleem@vt.edu](mailto:seleem@vt.edu)

**Table A.** Interaction between SQ109 and standard antifungal FLC on *Cryptococcus* isolates.

| Isolate ID | MIC (μg/mL) | | | | ΣFICI^a^ | Interpretation |
| --- | --- | --- | --- | --- | --- | --- |
|  | SQ109 | | FLC | |  |  |
|  | Alone | combined | alone | combined |  |  |
| *C. neoformans* H99 ATCC208821 | 2 | 0.5 | 4 | 1 | 0.5 | SYN |
| *C. neoformans* NR-41291 | 4 | 1 | 4 | 1 | 0.5 | SYN |
| *C. neoformans* NR-41292 | 4 | 2 | 8 | 1 | 0.63 | ADD |
| *C. neoformans* NR-41295 | 4 | 2 | 16 | 1 | 0.56 | ADD |
| *C. neoformans* NR-41296 | 4 | 1 | 4 | 1 | 0.5 | SYN |
| *C. neoformans*NR-41297 | 4 | 1 | 2 | 0.5 | 0.5 | SYN |
| *C. neoformans*NR-41298 | 4 | 1 | 4 | 0.5 | 0.38 | SYN |
| *C. neoformans*NR-41299 | 4 | 1 | 4 | 1 | 0.5 | SYN |
| *C. neoformans*NR-41300 | 4 | 2 | 1 | 0.5 | 1 | ADD |
| *C. neoformans* NR-50333 | 2 | 1 | 1 | 0.25 | 0.75 | ADD |
| *C. gattii* R265 NR-43208 | 4 | 1 | 4 | 1 | 0.5 | SYN |
| *C. gattii* CBS1930 NR-43209 | 4 | 2 | 8 | 1 | 0.63 | ADD |
| *C. gattii* NR-50422 | 4 | 1 | 16 | 4 | 0.5 | SYN |
| *C. gattii* NR-50423 | 4 | 1 | 8 | 2 | 0.5 | SYN |
| *C. gattii* NR-50425 | 4 | 1 | 8 | 2 | 0.5 | SYN |
| *C. gattii* NR-50426 | 4 | 1 | 4 | 0.5 | 0.38 | SYN |
| *C. gattii* NR-50427 | 2 | 1 | 4 | 2 | 1 | ADD |
| *C. gattii* NR-50429 | 4 | 2 | 2 | 0.25 | 0.63 | ADD |

^a^ Fractional inhibitory concentration index (ΣFICI); synergy (SYN) at FICI < 0.5, additive (ADD) at FICI between 0.5 and 1, indifferent at FICI between 1 and 2, and antagonistic at FICI > 4.

**Table B.** Box coordinates and best RMSD of redock poses

| Structure (PDB) | Box Coordinate | RMSD (Best, Å) |
| --- | --- | --- |
| 7WGH | (13, -51, -9) | 1.67 |
| 3WSB | (13, -51, -9) | 2.31 |
| 6C6P | (-8, -40, 0) | 0.66 |
| 4LXJ | (2, -58, -8) | 0.43 |


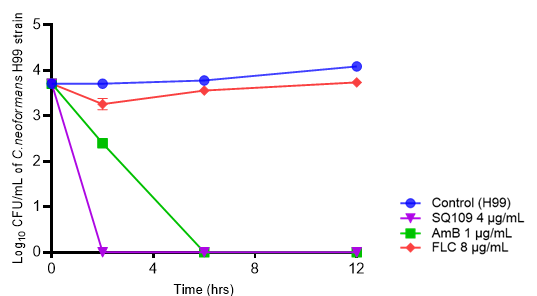


**Fig A. Effect of SQ109 on quiescent cryptococcal cells.** Independent inocula of C. neoformans H99 in PBS buffer (~5 x103 CFU/mL) were treated with SQ109 (4 µg/mL), alongside Amphotericin B (AmB) and fluconazole (FLC) as positive controls. The growth of the treated cells was monitored over a 48-hour period. Equal aliquots were diluted and spotted on yeast potato dextrose (YPD) agar at the corresponding time points.


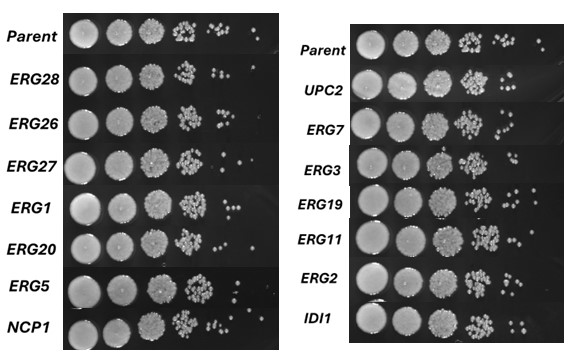


**Fig B**. Growth of heterozygous ergosterol mutants of *C. albicans* in RPMI-MOPS medium. Different strains were grown in RPMI-MOPS medium and incubated for 24 hrs, then serially diluted and plated on YPD agar.


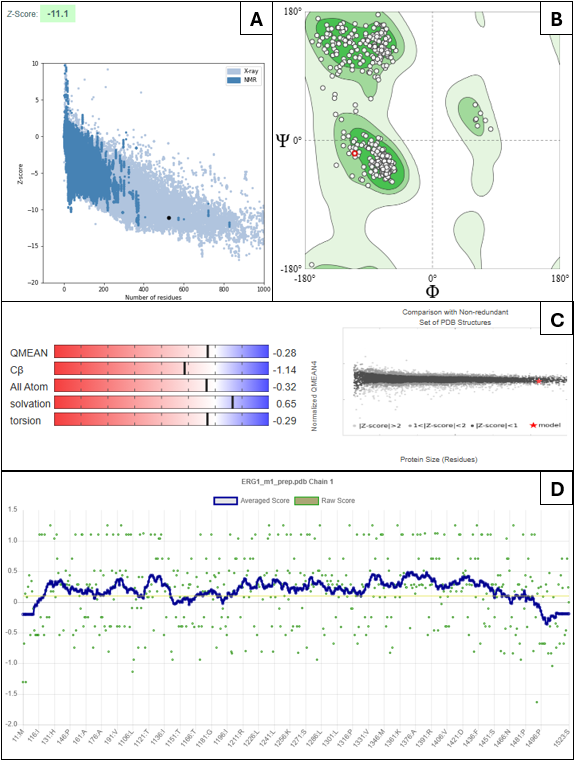


**Fig C.** Structural validation of the top-performing *C. neoformans* ERG1 AlphaFold3 model.


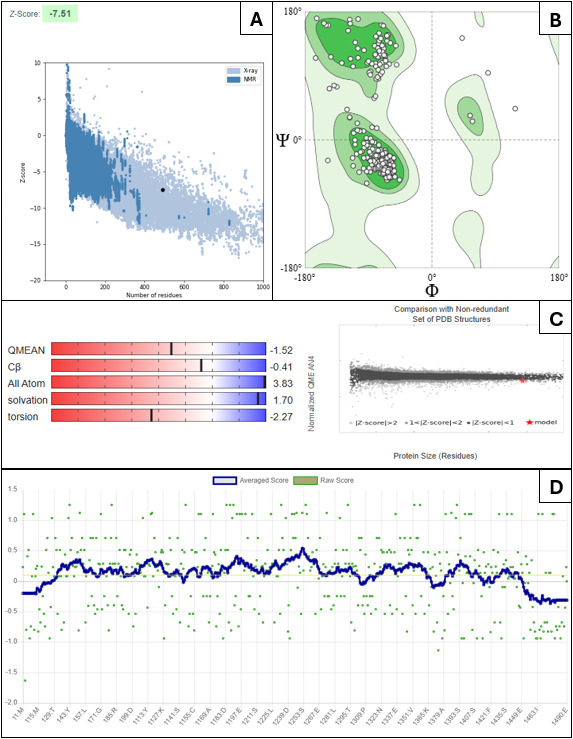


**Fig D.** Structural validation of the top-performing *C. neoformans* ERG9 AlphaFold3 model.


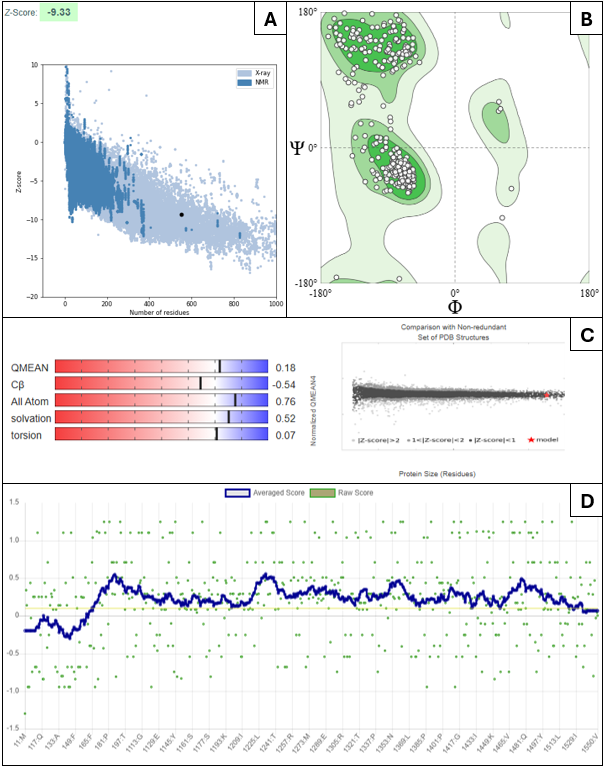


**Fig E.** Structural validation of the top-performing *C. neoformans* ERG11 AlphaFold3 model


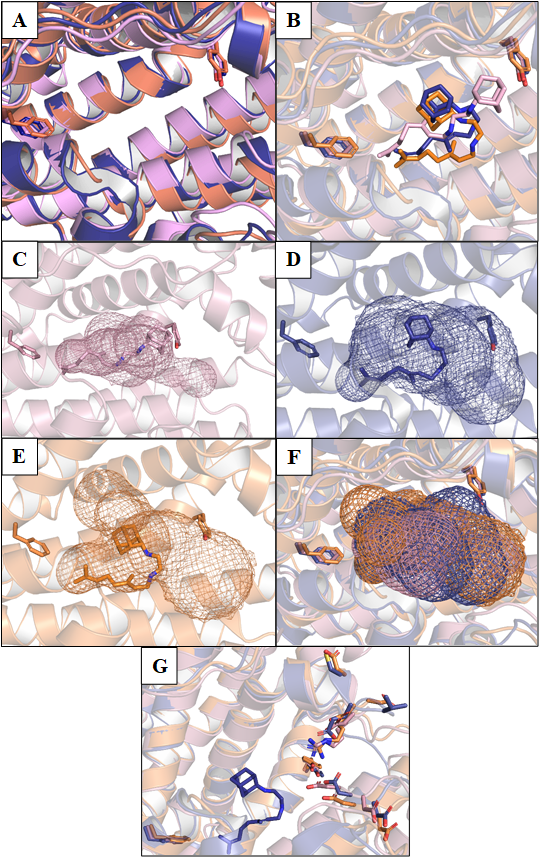


**Fig F.** ERG9 protein structures from *C. neoformans* (pink), *A. flavus* (orange), and *T. cruzi* (blue). **A**. Structural overlay of the squalene synthase active site with catalytic residues shown in sticks. **C-E**. Volumetric capacity of the squalene synthase binding cavity from *C. neoformans* (**C**), *T. cruzi* (**D**), and *A. flavus* (**E**) as defined using CAVER 3.0.3 with active site catalytic residues shown in sticks. **F.** Overlay of volumetric capacities from **C-E**. **G.** Crystal SQ109 in the active site with residues flanking the binding site entrance of *C. neoformans, T. cruzi, and A. flavus* shown in sticks.


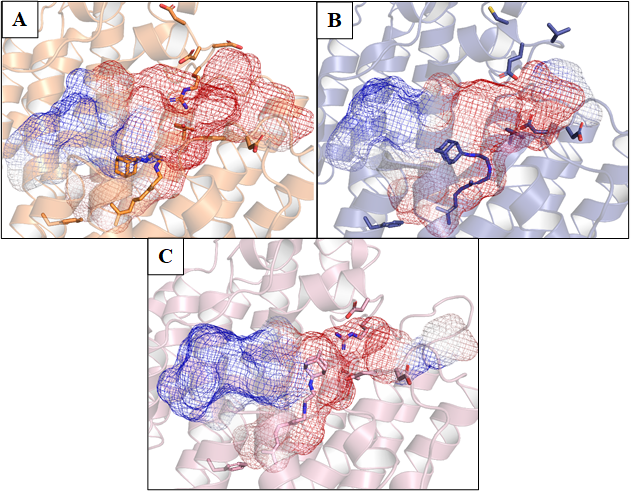


**Fig G.** Binding cavities of *A. flavus* (**A**)*, T. cruzi* (**B**)*, and C. neoformans* (**C**) colored by Columb potential, with red representing negative charge, blue representing positive charge, and white representing neutral charge. Side chains of residues flanking the binding site entrance are shown in sticks.


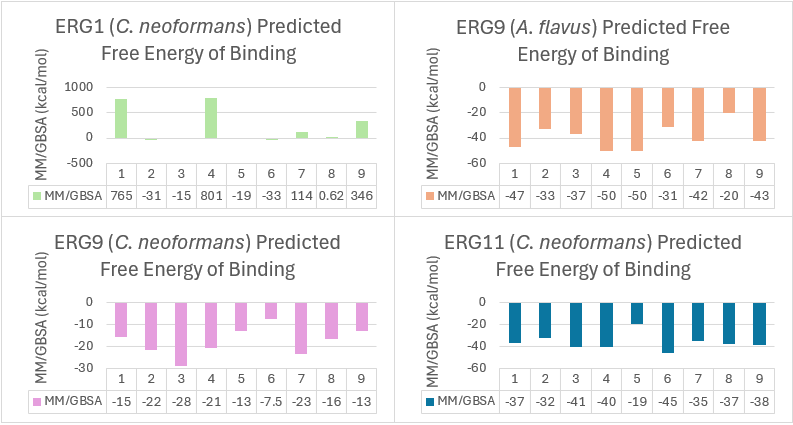


**Fig H.** MM/GBSA predicted free energy of binding of SQ109 docked poses calculated using Schrodinger Maestro 14.1.


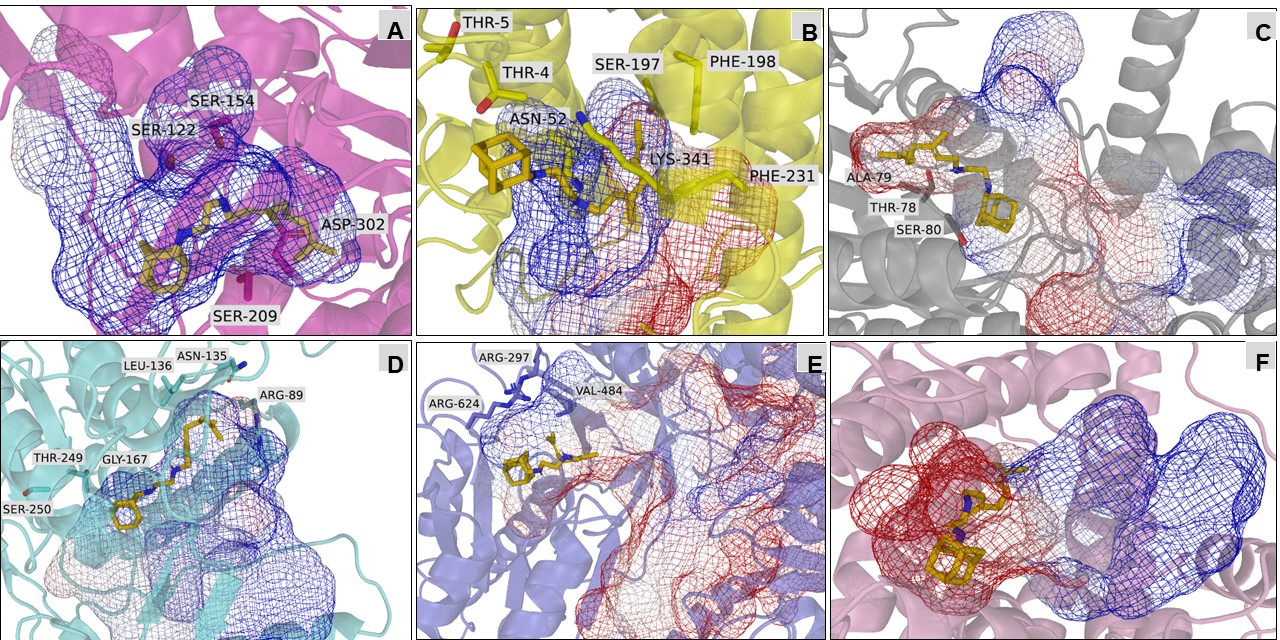


**Fig I.** Top poses of SQ109 (gold) in the binding sites of *ERG19* in magenta (**A**), *ERG20* in yellow (**B**), *ERG26* in gray (**C**), *ERG27* in teal (**D**), *NCP1* in blue (**E**), and *ERG9* in pink (**F**).
